# The relationships between interoception and alexithymic trait. The Self-Awareness Questionnaire in healthy subjects

**DOI:** 10.3389/fpsyg.2015.01149

**Published:** 2015-08-07

**Authors:** Mariachiara Longarzo, Francesca D'Olimpio, Angela Chiavazzo, Gabriella Santangelo, Luigi Trojano, Dario Grossi

**Affiliations:** ^1^Laboratory of Neuropsychology, Department of Psychology, Second University of NaplesCaserta, Italy; ^2^Hermitage CapodimonteNapoli, Italy

**Keywords:** interoceptive awareness, emotion, alexithymia, hypochondriasis, health, insular cortex

## Abstract

Interoception is the basic process enabling evaluation of one's own bodily states. Several previous studies suggested that altered interoception might be related to disorders in the ability to perceive and express emotions, i.e., alexithymia, and to defects in perceiving and describing one's own health status, i.e., hypochondriasis. The main aim of the present study was to investigate the relationships between alexithymic trait and interoceptive abilities evaluated by the “Self-Awareness Questionnaire” (SAQ), a novel self-report tool for assessing interoceptive awareness. Two hundred and fifty healthy subjects completed the SAQ, the Toronto Alexithymia Scale-20 items (TAS-20), and a questionnaire to assess hypochondriasis, the Illness Attitude Scale (IAS). The SAQ showed a two-factor structure, with good internal consistency (Cronbach's alpha = 0.88). We observed significant direct correlations between SAQ, TAS-20 and two of its subscales, and the IAS. Regression analysis confirmed that the difficulty in identifying and expressing emotions is significantly related with awareness for one's own interoceptive feelings and with a tendency to misinterpret and amplify bodily sensations. From a clinical point of view, the assessment of interoceptive awareness by the SAQ could be pivotal in evaluating several psychopathological conditions, such as the somatoform disorders.

## Introduction

Interoception is the basic process collecting information coming from one's own body, such as heartbeat, hunger, thirst, breathing, or visceral sensations. Such information is less distinct than that provided by “exteroceptive” somatosensory systems (e.g., touch, or skin temperature), but contributes to maintain homeostasis (Craig, 2003), and allows the brain to build a sense of one's own physical condition and to answer questions such as “how do you feel now?” (Craig, 2009).

The relationships between interoception and emotion are still under debate. Classic and modern theories of emotion maintain that bodily states contribute to or are essential for emotional experience (James, 1884; Lange, 1885; Bard, 1928; Cannon, 1932; Lane and Schwartz, 1987). Several studies using heartbeat perception tasks demonstrated a positive association between interoception and intensity of emotional experience (e.g., Schandry, 1981; Jones, 1994; Wiens et al., 2000; Barrett et al., 2004; Critchley et al., 2004; Wiens, 2005; Herbert et al., 2007; Pollatos et al., 2007a,b). Thus, according to Craig (2004, 2009), individual differences in emotional awareness may be directly related to individual differences in interoception. Similarly, Pollatos et al. (2005) and Dunn et al. (2010) reported that individuals with high sensitivity to interoceptive signals showed higher arousal in response to emotional visual stimuli.

The close relationships between interoception and emotional awareness have recently been supported by a neurofunctional study. Zaki et al. (2012) demonstrated that the anterior insular cortex, which is considered essential for both interoception monitoring and emotional processing (Craig, 2004, 2009; Critchley et al., 2004; Pollatos et al., 2007a), was activated during tasks in which healthy subjects were required to monitor their own heartbeat or to assess their own emotional experience after viewing videos of people recounting emotional stories. On this basis, the anterior insular cortex has been regarded as “a convergence zone” of interoceptive and emotional awareness (Zaki et al., 2012; see also Lamm and Singer, 2010). Enhanced activation in a network including the anterior insular cortex, the anterior cingulate cortex, ventromedial prefrontal cortex and somatosensory cortex has been reported in subjects with high interoceptive awareness (Critchley et al., 2004).

Emotional awareness and processing of interoceptive signals are thought to be impaired in alexithymia (Lane et al., 1998, 2000; Craig, 2004), defined by Sifneos (1973) as a reduced ability to identify and describe one's own emotions in patients with psychosomatic diseases. Alexithymia has been hypothesized to be associated with altered activation and morphology in the anterior cingulate cortex and the anterior insular cortex (Lane et al., 1998, 2000; Berthoz et al., 2002; Kano et al., 2003; Borsci et al., 2009). Consistent with such findings, Herbert et al. (2011) demonstrated that the ability to process interoceptive signals (assessed on a heartbeat perception task) is negatively associated with all aspects of alexithymia.

Available studies on the relationships between alexithymia and interoception, however, did not take into account the distinct facets of this complex construct. In this respect, Terasawa et al. (2013) underscored that tasks assessing heartbeat detection (e.g., Schandry, 1981) provide a measure of interoceptive “sensitivity,” i.e., of the accuracy in objective tests of bodily sensations, but are not suitable to investigate other aspects of interoception, such as interoceptive “sensibility” and interoceptive “awareness.” Interoceptive “sensibility” would express one's own tendency to be focused on internal states, whereas interoceptive “awareness” (IA) would imply cognitive appreciation of interoceptive sensations, and the ability of expressing bodily feelings. Interoceptive “sensibility” and IA are best addressed by means of self-report questionnaires (Terasawa et al., 2013). The distinction of the three aspects of interoception can help explaining the reason why studies using self-report questionnaires or “objective” heartbeat detection tasks provide divergent findings about the relationships between interoception and alexithymic trait. In fact, contrary to findings reported by Herbert et al. (2011), Ernst et al. (2014) observed a positive correlation between IA (assessed by a self-report tool, the Body Perception Questionnaire; Porges, 1993) and alexithymic trait in a small sample of healthy subjects in a study on neurotransmitter concentration in the insula and in the anterior cingulate cortex.

A positive correlation between IA and alexithymic trait could be consistent with the view that close attention toward interoceptive sensations could hamper interpretation of one's own emotional feelings (Biondi, 1991). Accordingly, in their review Kano and Fukudo (2013) suggested that persons with alexithymia tend to over-report physical symptoms and interpret even low-intensity emotion-related sensations as signs of illness. This hypothesis is consistent with the results of a PET study showing stronger awareness of visceral sensations in alexithymics, with greater activity in the posterior insula and in the rostral anterior cingulate cortex (Kano et al., 2007). Moreover, Tominaga et al. (2014) found a strong correlation between alexithymia (assessed by Toronto Alexithymia Scale-20 items; Bagby et al., 1994) and hypochondriasis and somatoform disorders (assessed by Somatosensory Amplification Scale; Barsky et al., 1988; Nakao and Barsky, 2007). Taken together, such observations would show a significant direct correlation between measures of IA and alexithymic trait, when self-report assessment tools are used.

The aim of the present study was to investigate the hypothesis of a direct association between IA and alexithymic trait in a large sample of healthy subjects. In a systematic review of the available self-report measures assessing body-awareness, Mehling et al. (2009) concluded that no instruments assess all aspects of interoception, but mainly explore anxiety or emotions, without sufficient details on physical sensations and with a few items on body awareness. For these reasons, Mehling et al. (2012) developed the Multidimensional Assessment of Interoceptive Awareness (MAIA) to evaluate dimensions of body awareness such as quality of body sensations, attention regulation for body sensations, emotional awareness for physiological signs of emotion or tendency to evaluate one's own body as safe and trustworthy. However, to the best of our knowledge, there is no tool evaluating perception of a wide range of bodily sensations. For this purpose, we devised a new self-report measure of IA to be used in a large non-clinical sample. This questionnaire, termed “Self-Awareness Questionnaire” (SAQ), is short and easy to administer and refers to commonly felt bodily sensations. This novel self-report tool is based on the “How do you feel questionnaire” (Grossi et al., 2014), which allowed to demonstrate that poor interoceptive awareness is associated with insular damage in stroke patients, and includes further items derived from other available questionnaires (e.g., Barsky et al., 1988; Porges, 1993).

## Materials and methods

### Participants

We recruited 250 healthy subjects among students and academic staff of the Departments of Psychology and Political Science at the Second University of Naples. To be included in the present study, subjects had to meet the following criteria: (1) lack of current or past history of alcohol or drug abuse, (2) lack of current or past history of major psychiatric diseases, (3) lack of history of brain injury, stroke, or any other major clinical condition, (4) lack of past or current use of psychoactive medications. The eligibility criteria were assessed by means of a brief semi-structured clinical interview.

All individuals were naïve to the scopes and purposes of the study and gave their written informed consent to participate without any reward.

### Materials and methods

All participants completed three questionnaires assessing: (i) IA, (ii) the ability to identify and describe emotions, and (iii) attitudes associated with hypochondriasis. To assess IA, and to specifically investigate how and how frequently subjects feel signals arising from their own body, we used an extended version of the “How do you feel questionnaire” (Grossi et al., 2014). The questionnaire included 35 items (Appendix 1) to be rated on a 5-point Likert scale (0 = never; 1 = sometimes; 2 = often; 3 = very often; 4 = always). The total score ranges 0–140, with higher scores meaning higher IA. In a preliminary study on an independent sample of 50 healthy students, we required participants to rate whether each item was clearly comprehensible and whether it assessed common physical sensations. All items were considered simple to comprehend, and suitable to address bodily sensations; all 35 items were thus included in the SAQ.

To assess the ability to identify and describe emotions, we used the Toronto Alexithymia Scale-20 items (TAS-20; Bagby et al., 1994), the most widely used self-report tool to assess the Alexithymia construct. The 20 items explore three factors reflecting the main aspects of the alexithymia: difficulty in identifying feelings; difficulty in describing feelings; externally oriented thinking. Each item has to be rated on a 5-point Likert scale (from 1 = “completely agree” to 5 = “strongly disagree”). The total score ranges 20–100, with higher scores indicating higher levels of alexithymia. The Italian version of TAS-20 has been demonstrated to show good test-retest reliability (0.86) and adequate internal consistency (Cronbach's alpha: 0.75) in a wide sample of healthy adults and of medical and psychiatric outpatients (Bressi et al., 1996).

The Illness Attitude Scale (IAS; Kellner, 1987) investigates attitude, fear and beliefs associated with hypochondriac behavior, and includes 27 items rated on a 5-point Likert scale (from 0 = “no” to 4 = “most of the time”). The total score ranges 0–108, with higher scores indicating more severe hypochondriac symptoms. The IAS is a reliable instrument, distinguishing hypochondriac patients from psychiatric patients and healthy individuals (Kellner, 1987). The IAS has been translated in several languages and its psychometric properties are well established (Sirri et al., 2008); an Italian version of the scale has been used in studies on clinical samples (e.g., Fava et al., 2000), but its psychometric properties have not been assessed specifically.

The study was approved by the Local Ethics Committees.

### Statistical analysis

Quality of data for SAQ questionnaire was evaluated by computing percentage of missing or invalid items; a percentage <5% is considered as an index of acceptable data quality. Moreover, data quality was assessed using mean, median, skewness, kurtosis and extent of ceiling and floor effects. Floor and ceiling effects <15% were defined as optimal (Cronbach, 1951). Internal consistency of the SAQ and of its dimensions was evaluated by Cronbach's alpha (McHorney and Tarlov, 1995); a value ≥0.70 was considered acceptable (Scientific Advisory Committee of the Medical Outcomes Trust, 2002). Scaling assumptions referring to the correct grouping of items were checked using alpha correction if item is removed (item is removed if the alpha value increases).

Furthermore, we run an Exploratory Factor Analysis, by Principal Axis Factor analysis (PAF), to explore the latent structure of the scale and to perform data reduction; we extracted the components explaining an amount of variance greater than a single item do (i.e., with Eigenvalues > 1; Kaiser, 1960). We computed factor loadings after oblimin rotation, allowing factors to correlate.

Thirdly, we assessed correlations of SAQ with TAS-20 (total and subscales scores for both measures) and IAS total score by computing Pearson's correlation coefficients (Bonferroni correction for multiple comparisons was adopted to reduce type-I errors).

Last, to investigate whether IA explained a significant portion of variance of alexithymia, a regression analysis was performed on TAS total score, using the SAQ total score as a predictor. The specific contribution of SAQ in predicting TAS scores was also assessed by means of hierarchical multiple regression analysis performed on TAS total scores, in which we first entered demographic data (age and gender), then IAS total score, and last SAQ total score as independent variables.

## Results

Two hundred and fifty healthy subjects (175 females, 75 males) participated in the present study. Table 1 shows the participants' demographic features and data about the psychometric variables.

**Table 1:** **Participants' demographic and psychometric variables**.

	**Mean**	**Standard deviation**	**Range**
Age (years)	27.9	9.4	18–57
Education (years)	14.3	2.3	5–18
IAS	33.6	15.4	4–81
TAS-20	34.0	9.8	9–69
SAQ	27.4	13.5	2–78

Data collected for each item of the SAQ were computable and there was no missing value. There was no floor or ceiling effect; skewness was 0.78 and kurtosis was 0.30.

Descriptive analyses on normality distribution and communality indexes from PAF, suggested removing 7 of the 35 items (Items: 1, 2, 5, 8, 20, 22, 29). The mean score on the resulting 28-item SAQ “awareness index” (max value = 112) for the present sample was 23.33 (*SD* = 11.67). PAF extracted three factors with an eigenvalue higher than 1. The screen test indicated a two-factor solution, which accounted for about 29% of the total variance. Factor loadings obtained after oblimin rotation are shown in Table 2.

**Table 2:** **Factor analysis of SAQ**.

**Items**	**F1**	**F2**
3. I feel my heart beat in my ears	**0.41**	0.21
4. I feel very hot in comparison to others	−0.16	**0.62**
6. I feel pain extremely	**0.32**	0.02
7. I feel my stomach tightening	**0.49**	0.18
9. I feel a sudden hunger pang	0.01	**0.47**
10. I feel my back ache	0.10	**0.32**
11. I feel pins and needles	0.19	**0.32**
12. I feel that I can't get enough air into my lungs	**0.72**	−0.09
13. I have an extra-strong heartbeat	**0.60**	0.13
14. I feel full and bloated after eating	0.21	**0.43**
15. I have the sudden urge to urinate	0.17	**0.36**
16. I feel as if I am on fire	0.23	**0.51**
17. I feel a burning sensation in my stomach	**0.32**	0.13
18. I feel a pain in my stomach	0.24	**0.30**
19. I feel very cold in comparison to others	**0.64**	−0.08
21. I feel like I have to throw up	**0.44**	0.17
23. I feel chilled	**0.60**	−0.10
24. I feel my legs heavy	0.25	**0.41**
25. I feel my throat dry	0.21	**0.47**
26. I have heavy feeling in my chest	**0.69**	−0.06
27. I feel my heart thudding	**0.71**	−0.10
28. I feel sudden thirst pangs	−0.10	**0.65**
30. I feel breathless without engaging in any type of exertion and effort	**0.32**	**0.31**
31. I feel my ears burning	0.14	**0.30**
32. I feel a lump in my throat	**0.63**	−0.05
33. I feel faint	**0.37**	0.07
34. I feel my palms sweaty	−0.14	**0.51**
35. I have difficulty in swallowing	**0.30**	0.07

*For each item the highest factor loading is printed in bold*.

The first factor (F1) was the most relevant (eigenvalue after rotation = 5.81), and mainly (but not exclusively) included items related to visceral sensations (items 3, 6, 7, 12, 13, 17, 19, 21, 23, 26, 27, 30, 32, 33, 35). The second factor (F2, eigenvalue = 4.50) mainly included items referring to somatosensory sensations (items 4, 9, 10, 11, 14, 15, 16, 18, 24, 25, 28, 30, 31, 34). The SAQ awareness index significantly correlated with both F1 (*r* = 0.89, *p* < 0.01) and F2 factors (*r* = 0.86, *p* < 0.01); the two factors significantly correlated with each other (*r* = 0.55, *p* < 0.01). The SAQ awareness index and its two factors showed good internal consistency (F1: alpha = 0.85; F2: alpha = 0.81; total alpha = 0.88). Correlation analyses (Table 3) revealed that the SAQ awareness index (and its two factors F1 and F2) significantly correlated with the TAS-20 total score and its two subscales “difficulty identifying feelings” and “difficulty describing feelings,” but not with the TAS-20 subscale assessing “externally oriented thinking.” In addition, the SAQ awareness index and its factors F1 and F2 significantly correlated with the IAS total score. All such correlations were positive, meaning that high scores on the SAQ and on its factors were associated to high scores on the questionnaires assessing two specific facets of alexithymia and hypochondriasis. By the same token, high IAS total scores were associated with high scores on TAS-20 and with high scores on two components of TAS-20, “difficulty identifying feelings” and “difficulty describing feelings.”

**Table 3:** **Correlation analyses between SAQ with IAS and TAS-20**.

	**TAS-20**				**IAS**
	**Difficulty identifying feelings**	**Difficulty describing feelings**	**Externally oriented thinking**	**Total score**	**Total score**
**SAQ** – Awareness index	0.47^**^	0.29^**^	−0.02	0.34^**^	0.32^**^
**SAQ –** F1 factor	0.40^**^	0.20^*^	−0.08	0.25^**^	0.31^**^
**SAQ** – F2 factor	0.45^**^	0.33^**^	0.04	0.37^**^	0.26^**^
**IAS** – Total score	0.36^**^	0.27^**^	0.10	0.33^**^	–

*Bonferroni corrected p-values*:

* *p < 0.05*;

** *p < 0.01*.

The results from the regression analysis showed that Interoception was a moderately significant predictor of Alexithymia, explaining 13% of the variance (beta = 0.37, *p* < 0.001). The results from the hierarchical regression models showed that: age and gender were not significant predictors of total TAS score [step# 1: *R2* = 0.002; *F*_(2, 247)_ = 1.28; *p* = 0.28; beta for age = 0.08, beta for gender = 0.06]; hypochondria was a significant predictor of alexithymia [step# 2: *R2* = 0.12; *F*_(3, 246)_ = 11.96; *p* < 0.001; beta = 0.35]; IA, as evaluated by the SAQ, was still a significant predictor of TAS scores accounting for a further 8% of the variance [step# 3: *R2* = 0.20; *F*_(4, 245)_ = 16.21; *p* < 0.001; beta for gender = 0.18, beta for IAS = 0.21, beta for SAQ = 0.33].

## Discussion

The aim of the present study was to investigate the relationships between bodily awareness, i.e., “interoceptive awareness” after Terasawa et al. (2013), and the ability to identify and to describe emotions. For this purpose we used a self-report questionnaire (SAQ) specifically assessing how and how frequently participants felt signals from their own body. The SAQ demonstrated a good internal consistency; items clustered into two factors, the first mainly related to visceral feelings, and the second mainly related to somatosensory feelings.

In exploring the relationships between IA and emotion processing, we observed significant positive relationships of SAQ awareness index and of its two factors with TAS-20. In particular, the two subscales of the TAS-20 investigating “difficulty in identifying feelings” and “difficulty in describing feelings” showed strong positive relationships with the SAQ awareness index and its two factors. It is important to underscore that the correlation of IA with alexithymic trait was positive in the present study, consistent with findings reported by Ernst et al. (2014), who observed that a high IA, as assessed by a self-report index, correlated with high alexithymic trait. Although studies assessing interoceptive “sensitivity” (e.g., via the heartbeat detection task) reported an inverse correlation (Herbert et al., 2011), our and Ernst et al.'s findings clearly support the idea that IA is directly correlated with alexithymic trait.

The association between hypochondriasis and interoception might suggest that high IA is related to a strong concern for one's own bodily sensations. Along these lines, Salkovskis and Warwick (1986) maintained that cognitive processes concerning body, health and illness might increase attention toward bodily signals. Barsky et al. (1990) subsumed this hypothesis in the Somatosensory Amplification model, according to which hypochondriac subjects tend to focus on their somatic sensations and experience them as intense and disturbing. This group of subjects are hypervigilant about their own body, concentrate on their own physical sensations and consider them as dangerous.

The significant positive correlations among the SAQ awareness index (and its two factors), TAS-20 scores and IAS total score suggest that focusing one's own attention on bodily feelings can hamper interpretation of one's own emotional feelings, and of bodily sensations that are direct expressions of such feelings. In other words, these results might be compatible with the idea that IA, emotion processing and hypochondriasis trait are different facets of a condition in which individuals focus on their own body signals and do not recognize the emotional nature of such signals. These individuals might be prone to interpret their physiological modifications as a cue that something life threatening is going on. Increased attention to one's own somatic signals might lead to a corresponding inattention to one's own emotions. These findings seem to confirm the interconnection between these conditions. However, from the present study we cannot infer the causal directionality of such correlations, and it remains entirely plausible that high scores of TAS-20 factors may contribute to the development of somatoform disorders through attention, amplification and misinterpretation of somatic sensations with emotional arousal (Kano and Fukudo, 2013).

It is important to take into account that our conclusions are based on observations gathered from a sample of relatively young and educated healthy adults, and this might limit generalization of our findings. However, most available scales, including the Italian version of the TAS-20 used in this study, have been developed and tested on similar samples. Moreover, in our study we did not assess validity and reliability of the SAQ questionnaire and did not investigate its relationships with objective measures of interoception (e.g., the heartbeat detection task). We also acknowledge that the inclusion of clinical samples would have added further informative values to the present study, allowing ascertaining whether the same relationships among IA, alexithymia and hypochondriasis also apply in individuals with somatoform disorders. These potential limitations might trigger further extensive investigation, in which the different aspects of interoception are assessed concurrently, and levels of anxiety and of depressive symptoms are controlled, since they could modulate alexithymia, and particularly the “difficulty identifying emotion” and “difficulty describing emotion” factors of TAS-20 (Tominaga et al., 2014).

Despite the above limitations, our study demonstrated relevant interactions between IA, alexithymia and hypochondriasis. IA is necessary for maintaining homeostasis, but excessive attention to the body can interfere with the ability to interpret bodily signals correctly. From a clinical point of view, the assessment of IA could be pivotal in evaluating several psychopathological conditions. Misinterpretation of physical sensations, rather than physical sensations in themselves, can determine concerns in individuals with a high level of attention to their own body; this could result in a vicious circle involving metacognition and evaluation of one's own thinking and symptoms (Marcus et al., 2007). These observations suggest that IA could contribute in maintaining such syndromes. The SAQ might reveal useful in comprehending the role of IA in clinical samples of patients with somatoform disorders.

### Conflict of interest statement

The authors declare that the research was conducted in the absence of any commercial or financial relationships that could be construed as a potential conflict of interest.

## Appendix

SELF-AWARENESS QUESTIONNAIRE (Original Italian version)

1. Mi capita che quando qualcuno tossisce, viene da tossire anche a me.
      (When somebody coughs, I feel like coughing too)
      □ never □ sometimes □ often □ very often □ always
2. Mi capita di sentire molto fastidio anche per una piccola ferita
      (I am excessively bothered even by a small wound)
      □ never □ sometimes □ often □ very often □ always
3. Mi capita di sentire il mio battito cardiaco pulsare nelle orecchie
      (I feel my heart beat in my ears)
      □ never □ sometimes □ often □ very often □ always
4. Mi capita di sentire eccessivamente caldo rispetto agli altri
      (I feel very hot in comparison to others)
      □ never □ sometimes □ often □ very often □ always
5. Mi capita di sentire la testa vuota
      (I feel my head empty)
      □ never □ sometimes □ often □ very often □ always
6. Mi capita di sentire eccessivamente il dolore
      (I feel pain excessively)
      □ never □ sometimes □ often □ very often □ always
7. Mi capita di sentire una stretta allo stomaco
      (I feel my stomach tightening)
      □ never □ sometimes □ often □ very often □ always
8. Mi capita di sentire un pizzicore alla gola
      (I feel a tickle in my throat)
      □ never □ sometimes □ often □ very often □ always
9. Mi capita di sentire un improvviso stimolo della fame
      (I feel a sudden hunger pang)
      □ never □ sometimes □ often □ very often □ always
10. Mi capita di sentire mal di schiena
      (I feel my back ache)
      □ never □ sometimes □ often □ very often □ always
11. Mi capita di sentire formicolii
      (I feel pins and needles)
      □ never □ sometimes □ often □ very often □ always
12. Mi capita di sentire che mi manca l'aria
      (I feel that I can't get enough air into my lungs)
      □ never □ sometimes □ often □ very often □ always
13. Mi capita di avere il batticuore
      (I have an extra-strong heartbeat)
      □ never □ sometimes □ often □ very often □ always
14. Mi capita di sentirmi gonfio dopo un pasto
      (I happen full and bloated after eating)
      □ never □ sometimes □ often □ very often □ always
15. Mi capita di sentire un improvviso stimolo della pipì
      (I have a sudden urge to urinate)
      □ never □ sometimes □ often □ very often □ always
16. Mi capita di sentirmi infuocare
      (I feel as if I am on fire)
      □ never □ sometimes □ often □ very often □ always
17. Mi capita di sentire bruciore di stomaco
      (I feel a burning sensation in my stomach)
      □ never □ sometimes □ often □ very often □ always
18. Mi capita di sentire mal di pancia
      (I feel a pain in my stomach)
      □ never □ sometimes □ often □ very often □ always
19. Mi capita di sentire eccessivamente freddo rispetto agli altri
      (I feel very cold in comparison to others)
      □ never □ sometimes □ often □ very often □ always
20. Mi capita di sentire prurito
      (I feel itchy)
      □ never □ sometimes □ often □ very often □ always
21. Mi capita di sentire che devo vomitare
      (I feel as if I have to throw up)
      □ never □ sometimes □ often □ very often □ always
22. Mi capita di sentire un improvviso stimolo della cacca
      (I happen a sudden urge to defecate)
      □ never □ sometimes □ often □ very often □ always
23. Mi capita di sentirmi agghiacciare
      (I feel chilled)
      □ never □ sometimes □ often □ very often □ always
24. Mi capita di sentire le gambe pesanti
      (I feel my legs are heavy)
      □ never □ sometimes □ often □ very often □ always
25. Mi capita di sentire la gola secca
      (I feel my throat dry)
      □ never □ sometimes □ often □ very often □ always
26. Mi capita di sentire un peso al petto
      (I have a heavy feeling in my chest)
      □ never □ sometimes □ often □ very often □ always
27. Mi capita di sentire un tonfo al cuore
      (I feel my heart thudding)
      □ never □ sometimes □ often □ very often □ always
28. Mi capita di sentire un improvviso stimolo della sete
      (I feel sudden thirst pangs)
      □ never □ sometimes □ often □ very often □ always
29. Mi capita di sentire un dolore che si sposta da una parte all'altra del corpo
      (I feel a pain that seems to migrate around the body)
      □ never □ sometimes □ often □ very often □ always
30. Mi capita di sentire l'affanno senza aver fatto alcuno sforzo
      (I feel breathless without engaging in any type of exertion or effort)
      □ never □ sometimes □ often □ very often □ always
31. Mi capita di sentire le orecchie bollenti
      (I feel my ears burning)
      □ never □ sometimes □ often □ very often □ always
32. Mi capita di sentire un nodo alla gola
      (I feel a lump in my throat)
      □ never □ sometimes □ often □ very often □ always
33. Mi capita di sentirmi svenire
      (I feel faint)
      □ never □ sometimes □ often □ very often □ always
34. Mi capita di sentire le mani sudate
      (I feel my palms sweaty)
      □ never □ sometimes □ often □ very often □ always
35. Mi capita di sentire difficoltà ad ingoiare
      (I have difficulty swallowing)
      □ never □ sometimes □ often □ very often □ always

ISTRUZIONI.

Per cortesia legga attentamente ogni domanda e scelga la risposta che meglio descrive quanto spesso avverte ogni sensazione. Indichi una sola risposta. Non ci sono risposte corrette o errate.

(Please read each item carefully and tick the one box that best describes how often you feel each sensation. Tick one only. There are no right or wrong answers.)
